# Increased GABA_B_ receptor signaling in a rat model for schizophrenia

**DOI:** 10.1038/srep34240

**Published:** 2016-09-30

**Authors:** Martijn M. Selten, Francisca Meyer, Wei Ba, Astrid Vallès, Dorien A. Maas, Moritz Negwer, Vivian D. Eijsink, Ruben W. M. van Vugt, Josephus A. van Hulten, Nick H. M. van Bakel, Joey Roosen, Robert J. van der Linden, Dirk Schubert, Michel M. M. Verheij, Nael Nadif Kasri, Gerard J. M. Martens

**Affiliations:** 1 Department of Cognitive Neuroscience, Radboud University Medical Center, Donders Institute for Brain, Cognition, and Behaviour, Nijmegen, the Netherlands; 2 Department of Molecular Animal Physiology, Donders Institute for Brain, Cognition and Behaviour, Centre for Neuroscience, Radboud University, Nijmegen, the Netherlands; 3 Department of Human Genetics, Radboud University Medical Center, Donders Institute for Brain, Cognition and Behaviour, Nijmegen, the Netherlands; 4 Department of Cognitive Neuroscience, Faculty of Psychology and Neurosciences, Maastricht University, Maastricht, the Netherlands; 5 Department of Language and Genetics, Max Planck Institute for Psycholinguistics, Nijmegen, the Netherlands

## Abstract

Schizophrenia is a complex disorder that affects cognitive function and has been linked, both in patients and animal models, to dysfunction of the GABAergic system. However, the pathophysiological consequences of this dysfunction are not well understood. Here, we examined the GABAergic system in an animal model displaying schizophrenia-relevant features, the apomorphine-susceptible (APO-SUS) rat and its phenotypic counterpart, the apomorphine-unsusceptible (APO-UNSUS) rat at postnatal day 20–22. We found changes in the expression of the GABA-synthesizing enzyme GAD67 specifically in the prelimbic- but not the infralimbic region of the medial prefrontal cortex (mPFC), indicative of reduced inhibitory function in this region in APO-SUS rats. While we did not observe changes in basal synaptic transmission onto LII/III pyramidal cells in the mPFC of APO-SUS compared to APO-UNSUS rats, we report reduced paired-pulse ratios at longer inter-stimulus intervals. The GABA_B_ receptor antagonist CGP 55845 abolished this reduction, indicating that the decreased paired-pulse ratio was caused by increased GABA_B_ signaling. Consistently, we find an increased expression of the GABA_B1_ receptor subunit in APO-SUS rats. Our data provide physiological evidence for increased presynaptic GABA_B_ signaling in the mPFC of APO-SUS rats, further supporting an important role for the GABAergic system in the pathophysiology of schizophrenia.

Schizophrenia is a complex mental disorder thought to result from a combination of genetic and environmental factors that provide vulnerability to early- and later-life stressors^1^,^2^,^3^. Growing evidence suggests that schizophrenia may arise from abnormal connectivity between various integrative brain regions, especially the prefrontal cortex (PFC), the medial temporal lobe, and striatal regions^4^,^5^,^6^,^7^. For several decades, schizophrenia research was guided by the dopamine hypothesis^8^. However, this hypothesis has expanded more recently into a view including other neurotransmitters such as serotonin^9^, glutamate^10^ and γ-aminobutyric acid (GABA)^11^.

Dysfunction of the GABAergic system in schizophrenia has been well documented by postmortem studies showing alterations in a number of pre- and post-synaptic markers of inhibitory neurotransmission thought to be most prominent in parvalbumin positive (PV^+^) neurons^12^. For example, reduced densities of calbindin-positive (CB^+^) and PV^+^ GABAergic interneurons have been observed in the PFC of schizophrenia patients^13^,^14^,^15^. In addition, protein levels of GABA transporter 1 (GAT1) are reduced in the PFC of schizophrenia patients^16^. Also, PFC levels of the GABA synthesizing enzyme GAD67 are reduced in schizophrenia, which can be attributed to reduced expression in PV^+^ cells specifically^17^ and is indicative of reduced GABAergic signaling^18^. At the synaptic level, the GABAAα2 subunit, which is prominently expressed in synapses formed by chandelier cells on the axon initial segment of pyramidal neurons, is upregulated in schizophrenia patients^19^. In line with these results, a genetic model of schizophrenia, the Erbb4 knock-out mouse, shows changes in GABAAα2 and GAT1 density as well as a reduced number of inhibitory synapses on excitatory pyramidal cells^12^,^20^. Conversely, excitatory inputs onto PV^+^ cells are decreased in these mice^12^,^20^.

To explore the pathophysiological mechanisms underlying schizophrenia, we used the apomorphine-susceptible (APO-SUS) rat model and its phenotypic counterpart, the apomorphine-unsusceptible (APO-UNSUS) rat. These two rat lines have been pharmacogenetically selected and bred from an outbred Wistar population based on their stereotypical gnawing responses to the dopamine D1/D2 agonist apomorphine^21^. The APO-SUS rat is a well-characterized animal model that displays schizophrenia-relevant features during adulthood, such as altered density of central dopamine receptors^22^, high sensitivity to dopaminergic drugs (i.e., apomorphine and amphetamine)^21^,^23^,^24^, decreased prepulse inhibition and diminished latent inhibition^24^,^25^, increased novelty-induced exploration and accumbal dopamine response^21^,^23^,^26^, and an increased HPA-axis response to stress^22^, as well as learning and memory deficits^27^,^28^. However, the pathophysiology underlying the phenotypes observed in this model remains unknown.

In the present study, we compared the inhibitory system in the medial PFC (mPFC) of APO-SUS and APO-UNSUS rats at postnatal day (PND) 20–22. We found a decrease in the protein level of GAD67 as well as a reduced cell-count for GAD67^+^ cells specifically in the prelimbic region (PRL) of the mPFC. While basal synaptic transmission onto LII/III pyramidal cells was not different between the two rat lines, we report a decrease in the paired-pulse ratio in APO-SUS rats at longer (≥150 ms) intervals compared to APO-UNSUS rats. Importantly, this decrease could be abolished by application of the GABA_B_ antagonist CGP 55845, indicating that the decreased paired-pulse ratio was caused by an increased activity in GABA_B_ signaling. We indeed observed an increase in GABA_B_ receptor expression in the mPFC of APO-SUS rats compared to APO-UNSUS rats. Collectively, our data identify GABA_B_ receptor signaling as a possible player in the etiology of schizophrenia.

## Results

### Reduced levels of GAD67 in the mPFC of APO-SUS rats

**Figure 1:**
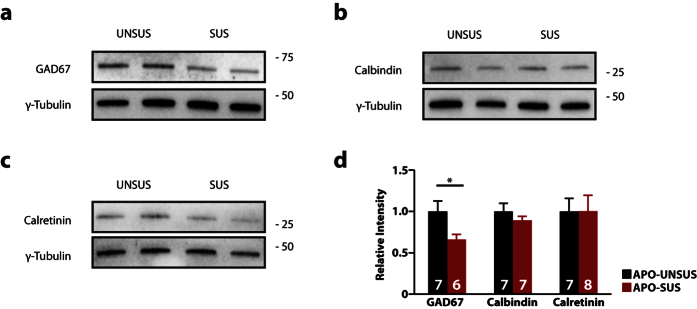
Reduced expression of GAD67, but not Calbindin (CB) and Calretinin (CR) in the medial prefrontal cortex (mPFC) of APO-SUS rats. (**a–c**) Representative immunoblot images for GAD67 (**a**), CB (**b**) and CR **(c,d)** quantification of western blot analysis for CB, CR and GAD67 in APO-UNSUS and APO-SUS mPFC. Expression is normalized to APO-UNSUS expression of the respective protein. Sample size (n) is indicated in the bars. Data is presented as mean ± SEM, * p < 0.05. Student’s t-test.

Previous studies have reported altered levels of specific interneuron markers in schizophrenia patients^13^,^14^,^15^,^16^,^17^,^29^. In order to determine whether the number of GABAergic interneurons is altered in APO-SUS compared to APO-UNSUS rats, we performed western blot analysis to measure the protein expression levels of GAD67, CB and CR in punches from the mPFC of PND 20–22 rats (Fig. 1). We found protein levels of GAD67 to be reduced by ~35% in APO-SUS rats (Fig. 1a,d; p = 0.04). Conversely, we found no change in the levels of CB (Fig. 1b,d; p = 0.33) and CR (Fig. 1c,d; p = 0.99) between the two rat lines.

### Reduction of GAD67^+^ cells in the prelimbic region, but not the infralimbic region, of the mPFC of APO-SUS rats

Next, we wanted to assess whether the decrease in GAD67 protein levels in the mPFC was due to a decreased amount of GAD67 per cell or to a decrease in the total number of interneurons. To this end we performed immunohistochemistry to assess the number of interneurons positive for GAD67. We also assessed the number of CB^+^, CR^+^ and PV^+^ cells, which together label ~90% of GABAergic interneurons^30^. We used coronal sections of the mPFC and analyzed the number of positive cells separately for the prelimbic (PRL)- and infralimbic (IL) region, two subregions of the mPFC.

**Figure 2:**
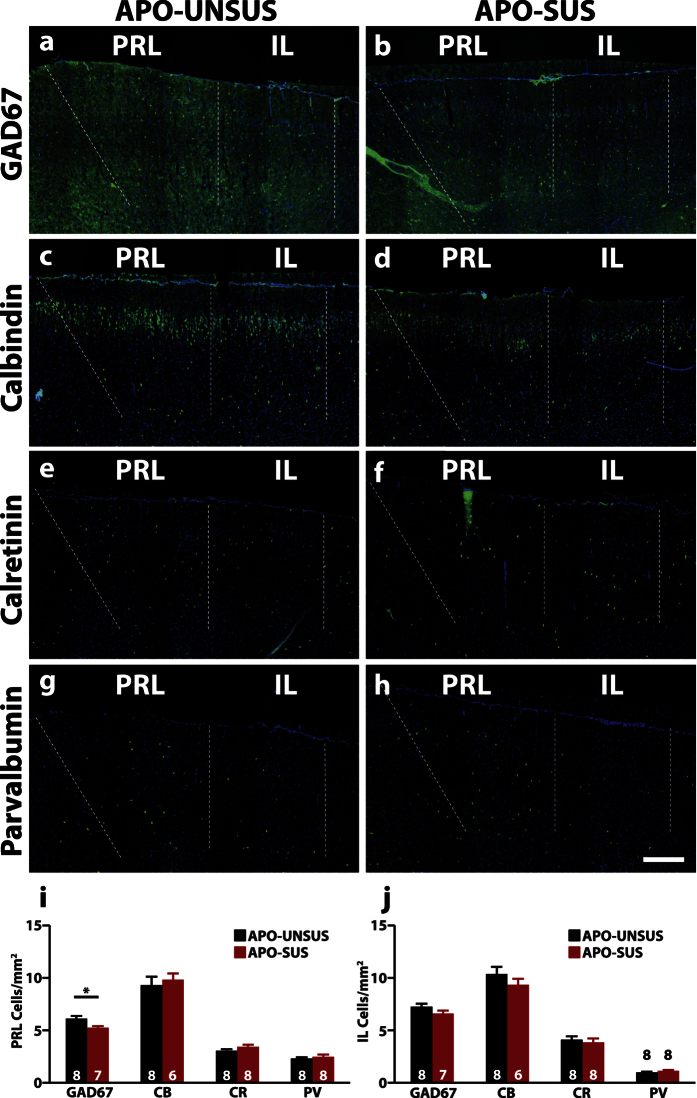
Reduced number of GAD67^+^ cells in the PRL, but not the IL. Representative images of a staining for GAD67 (**a,b**), Calbindin (CB) (**c,d**) Calretinin (CR) (**e,f**) and parvalbumin (PV) (**g,h**) and quantification in the prelimbic (PRL) (**i**) and infralimbic (IL) region (**j**) of the medial prefrontal cortex. GAD67, CB, CR and PV staining in green, Hoechst in blue. Sample size (n) is indicated in the bars. Scale bar: 200 μm. Data is presented as mean ± SEM. **p < 0.01. Student’s t-test.

We found a significant decrease of GAD67^+^ cells specifically in the PRL (APO-UNSUS 6.12 ± 0.25 cells/mm^2^; APO-SUS 5.25 ± 0.15 cells/mm^2^, p = 0.012; Fig. 2a,b,i), but not the IL (APO-UNSUS 7.25 ± 0.29 cells/mm^2^; APO-SUS 6.60 ± 0.28 cells/mm^2^, p = 0.13; Fig. 2a,b,j) in APO-SUS rats. We found no changes in the levels of CB^+^, CR^+^ and PV^+^ cells in both the PRL (CB^+^: APO-UNSUS 9.32 ± 0.79 cells/mm^2^; APO-SUS 9.82 ± 0.60 cells/mm^2^, p = 0.64; CR^+^: APO-UNSUS 3.06 ± 0.16 cells/mm^2^; APO-SUS 3.43 ± 0.22 cells/mm^2^, p = 0.19; PV^+^: APO-UNSUS 2.32 ± 0.11 cells/mm^2^; APO-SUS 2.49 ± 0.21 cells/mm^2^, p = 0.47), or IL (CB^+^: APO-UNSUS 10.36 ± 0.70 cells/mm^2^; APO-SUS 9.33 ± 0.59 cells/mm^2^, p = 0.33; CR^+^: APO-UNSUS 4.12 ± 0.32 cells/mm^2^; APO-SUS 3.85 ± 0.37 cells/mm^2^, p = 0.58; PV^+^: APO-UNSUS 0.99 ± 0.08 cells/mm^2^; APO-SUS 1.13 ± 0.11 cells/mm^2^, p = 0.30; Fig. 2c–j). The fact that we did not observe changes in the CB^+^ CR^+^ and PV^+^ populations, which suggests the reduction in GAD67 is due to a reduced level of GAD67 per cell, rather than a reduction in the total amount of interneurons, specifically in the PRL.

### Unaltered basal synaptic input on LII/III pyramidal neurons in the mPFC

**Figure 3:**
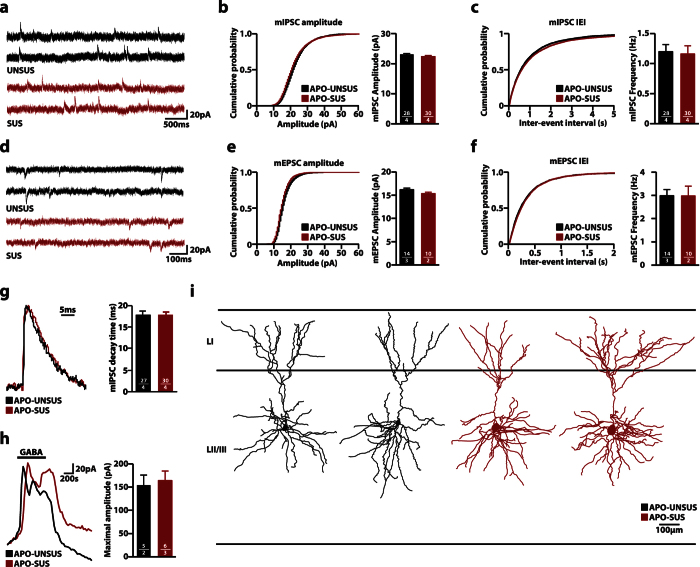
No changes in basal synaptic transmission and morphology in LII/III pyramidal cells in the medial prefrontal cortex (mPFC). (**a**) Representative traces of miniature inhibitory post-synaptic currents (mIPSCs) recorded from LII/III pyramidal cells from acute brain slices from APO-UNSUS and APO-SUS rats. (**b,c**) Cumulative distributions of mIPSC amplitudes (**b**) and inter-event intervals (IEI) **(c)** from APO-UNSUS and APO-SUS rats. (**d**) Representative traces of miniature excitatory post-synaptic currents (mEPSCs) recorded from LII/III pyramidal cells from acute brain slices from APO-UNSUS and APO-SUS rats **(e,f)** Cumulative distributions of mEPSC amplitudes **(e)** and inter-event intervals (**f**) from APO-UNSUS and APO-SUS rats. (**g**) Representative traces (scaled) and distribution of mIPSC decay time. (**h**) Representative traces and quantification of systemic GABA (20 μM) application from APO-UNSUS and APO-SUS rats. Black bar indicates GABA application. (**i**) Representative reconstructions of LII/III pyramidal neurons from mPFC. Sample size (n) is indicated in the bars as number of cells/number of animals. Bar graphs are presented as mean ± SEM. Student’s t-test.

Since reduced levels of GAD67 are indicative of reduced interneuronal activity^31^,^32^, we subsequently tested if the observed changes in GAD67 protein levels would be accompanied by changes at the electrophysiological level. In order to assess basal synaptic input we used whole-cell voltage clamp to record miniature inhibitory postsynaptic currents (mIPSCs) from layer (L) II/III pyramidal neurons in the mPFC (Fig. 3a–c). We found no difference in both mIPSC amplitude (APO-UNSUS 23.04 ± 0.32 pA; APO-SUS 22.33 ± 0.37 pA, p = 0.16) and frequency (APO-UNSUS 1.20 ± 0.12 Hz; APO-SUS 1.16 ± 0.14 Hz, p = 0.83). Similarly, no differences were found in miniature excitatory postsynaptic currents (mEPSCs) between the two rat lines (amplitude: APO-UNSUS 16.20 ± 0.31 pA; APO-SUS 15.34 ± 0.31 pA; p = 0.08; frequency: APO-UNSUS 2.99 ± 0.26 Hz; APO-SUS 2.98 ± 0.42 Hz, p = 0.98) (Fig. 3d–f).

Previous studies have shown that changes in postsynaptic GABA receptor subunit composition are reflected in the decay time of mIPSC^33^,^34^. We found no changes in the mIPSC decay time in APO-SUS rats (APO-UNSUS 15.58 ± 0.52 ms; APO-SUS 13.46 ± 0.93 ms, p = 0.06), indicating the postsynaptic receptor content of inhibitory synapses was unaltered (Fig. 3g).

Next, we measured the maximal response to bath application of 20 μM GABA (Fig. 3h). No difference was observed between APO-UNSUS and APO-SUS rats (APO-UNSUS 154.1 ± 48.3 pA; APO-SUS 163.9 ± 51.2 pA, p = 0.78), indicating that the total number of GABA_A_ receptors was identical. Together, these data show that there is no difference in basal synaptic inhibitory and excitatory input connectivity onto LII/III pyramidal cells in the mPFC between APO-UNSUS and APO-SUS rats.

We then analyzed pyramidal cell morphology of cells that were filled with biocytin during the recordings. All cells had a typical pyramidal morphology, hallmarked by a skirt of basal dendrites radiating ~200 μm from the soma, and a single long apical dendrite that reached into upper LI (Fig. 3i). The apical dendrite mostly branched close to the soma, and had an apical tuft close to the pia mater, mainly within LI, consistent with previous reports^35^. The dendritic morphology of both apical and basal dendrites did not differ between pyramidal cells from APO-UNSUS and APO-SUS rats (Table 1).

### Reduction in paired-pulse ratio through GABA_B_ receptor signaling in the mPFC of APO-SUS rats

**Figure 4:**
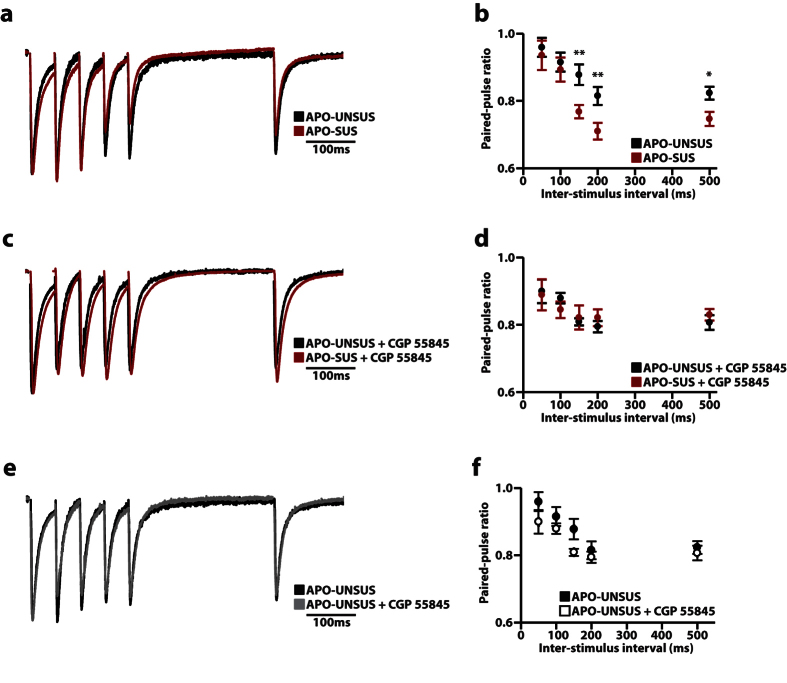
APO-SUS rats show a reduced paired-pulse ratio at long ≥ 150 ms inter-stimulus intervals (ISI). (**a,b**) Representative traces (**a**) and quantification (**b**) of the paired-pulse ratio at different ISIs from APO-UNSUS (n = 14/3) and APO-SUS (n = 15/4) rats. **(c,d)** Representative traces **(c)** and quantification (**d**) of the paired-pulse ratio at different ISIs in the presence of the GABA_B_ antagonist CGP 55845 from APO-UNSUS (n = 10/3) and APO-SUS (n = 12/3) rats. (**e,f**) Comparison between APO-UNSUS in the absence or presence of CGP 55845, note that these traces are the same as in **a–d**. Representative traces show an overlay of all 5 individual inter-stimulus intervals. Sample size (n) is shown as number of cells/number of animals. Data are presented as mean ± SEM. *p < 0.05; **p < 0.01. Student’s t-test with Holm-Sidak correction for multiple comparisons.

Next, we investigated the release probability of inhibitory synapses onto LII/III pyramidal cells by recording the paired-pulse ratio following stimulation in the same layer in the PFC. We recorded paired-pulse ratios at different inter-stimulus intervals (ISI) (Fig. 4a,b) and found that the paired-pulse ratio were indistinguishable between both rat lines for short ISIs (≤100 ms) (50 ms APO-UNSUS 0.96 ± 0.03; APO-SUS 0.94 ± 0.04, p = 0.67; 100 ms APO-UNSUS 0.92 ± 0.03; APO-SUS 0.89 ± 0.04, p = 0.65), whereas LII/III pyramidal cells from APO-SUS rats showed a reduced paired-pulse ratio at longer ISIs (≥150 ms) (150 ms APO-UNSUS 0.88 ± 0.03; APO-SUS 0.77 ± 0.02; p < 0.01; 200 ms APO-UNSUS 0.81 ± 0.03; APO-SUS 0.71 ± 0.02; p < 0.01; 500 ms APO-UNSUS 0.82 ± 0.02; APO-SUS 0.75 ± 0.02; p < 0.05; Fig. 4b). Whereas changes at short ISIs are indicative of an altered release probability, changes in paired-pulse ratios for longer ISIs can be indicative of reduced presynaptic calcium inflow^36^. Previous studies have shown that a reduced presynaptic calcium inflow can be caused by increased retrograde signaling via nitric oxide^37^ and endocannabinoids^38^, or through binding of GABA to presynaptic GABA_B_ receptors, which upon activation negatively influence Ca^2+^ inflow through voltage gated Ca^2+^-channels^39^. Since only GABA_B_ receptors act at the sub-second time range, we investigated the involvement of GABA_B_ receptor signaling by recording the paired-pulse ratio in the presence of the GABA_B_ receptor antagonist CGP 55845. In APO-SUS rats, the paired-pulse ratio at 50 ms and 100 ms was unaffected by CGP 55845, consistent with GABA_B_ receptor activation time^40^. Interestingly, we found that the reduced paired-pulse ratio at longer ISIs (150, 200 and 500 ms) was completely abolished in the presence of CGP 55845 (50 ms APO-UNSUS 0.90 ± 0.03; APO-SUS 0.89 ± 0.05, p = 0.75; 100 ms APO-UNSUS 0.88 ± 0.02; APO-SUS 0.84 ± 0.02, p = 0.29; 150 ms APO-UNSUS 0.81 ± 0.01; APO-SUS 0.82 ± 0.04, p = 0.68; 200 ms APO-UNSUS 0.79 ± 0.02; APO-SUS 0.82 ± 0.03, p = 0.38; 500 ms APO-UNSUS 0.81 ± 0.02; APO-SUS 0.83 ± 0.02, p = 0.24; Fig. 4c,d), indicating that increased GABA_B_ receptor signaling underlies the decreased paired-pulse ratio in APO-SUS compared to APO-UNSUS rats. Of note, blocking of GABA_B_ receptors with CGP 55845 had no effect on the paired-pulse ratio of APO-UNSUS rats at all ISIs tested (Fig. 4e,f), indicating that GABA_B_ receptor signaling does not play a role in these experiments in APO-UNSUS rats.

### Increased expression of GABA_B_ receptors, but not GAT1, in the mPFC of APO-SUS rats

**Figure 5:**
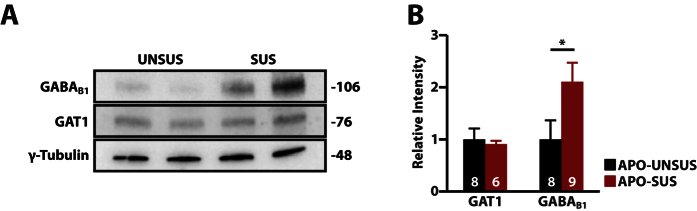
The expression of GABA_B1_, but not GAT1, is increased in the medial prefrontal cortex of APO-SUS rats. (**a**) Representative blot and (**b**) quantification of western blot analysis for GAT1 and GABA_B1_ on APO-UNSUS and APO-SUS. Expression is normalized to APO-UNSUS expression of the respective protein. Sample size (n) is indicated in the bars. Data is presented as mean ± SEM, *p < 0.05. Student’s t-test.

The increase in GABA_B_ receptor signaling could be caused either by an increase of GABA_B_ receptor activation, through increased levels of extracellular GABA, or by an increase in the number of GABA_B_ receptors. Increased levels of extracellular GABA could be caused by a reduction in the expression of GAT1, the main GABA transporter in the central nervous system^41^. GAT1 reduces the amount of extracellular GABA in the synapse, which reduces spillover^41^. GABA_B_ receptors are located perisynaptically, and are activated by GABA spillover. In addition, reduced GAT1 expression has been found in schizophrenia patients^42^ and animal models^12^ of schizophrenia. We therefore first assessed the levels of GAT1 in punches from the mPFC of APO-SUS and APO-UNSUS rats by performing western blot analysis. However, we found no significant difference between APO-SUS and APO-UNSUS rats (p = 0.73; Fig. 5a,b). We next studied the protein expression levels of GABA_B_ receptors. Western blot analysis using an antibody against the GABA_B1_ subunit, which together with the GABA_B2_ receptor forms an active GABA_B_ receptor^43^, revealed that the levels of GABA_B1_ were increased by ~110% in the mPFC of APO-SUS rats compared to APO-UNSUS mPFC (p < 0.05; Fig. 5a,b). Together, our data suggest that increased GABA_B_ receptor signaling in APO-SUS rats is accompanied by increased expression of the GABA_B1_ subunit, and underlies the decreased paired-pulse ratio in APO-SUS rats.

## Discussion

The GABAergic system plays an important role in the pathophysiology of schizophrenia^44^,^45^. Here, using the APO-SUS/APO-UNSUS rat model at PND 20–22 we further highlight the importance of dysfunctional GABA signaling in schizophrenia-related traits. We provide several lines of evidence for deficits in the inhibitory circuitry within the PRL of APO-SUS rats: (i) a reduced level of GAD67 in interneurons in the mPFC of APO-SUS rats, specifically in the PRL; (ii) a reduced paired-pulse ratio at longer (≥150 ms) ISIs in APO-SUS rats; and (iii) increased GABA_B_ signaling due to an increased expression of the GABA_B1_ subunit of the GABA_B_ receptor. Finally, we were able to reverse the increased GABA_B_ signaling in APO-SUS rats by the application of the GABA_B_ antagonist CGP 55845.

In our present study on the GABAergic system in the APO-SUS rats, we observed a decreased protein level of GAD67 in the PRL. Also, we found a reduced number of GAD67^+^ cells in this region. However, the number of CB^+^, CR^+^ and PV^+^ neurons, which together account for ~90% of interneurons^30^, remained unchanged in the APO-SUS rat, indicating that the total number of interneurons is unaltered. The reduced number of GAD67^+^ cells could result from a reduction of GAD67 levels per cell, causing these interneurons to be under the detection threshold of our analysis. Indeed, reduced levels of GAD67 have been reported before in schizophrenia both in animal models and post-mortem patient studies^17^,^46^,^47^.

A number of studies has shown that reduced prefrontal inhibitory transmission induces various cognitive, emotional and dopaminergic abnormalities that resemble aspects of schizophrenia^48^. More specifically, reduced GABAergic activity in the PRL of the rat PFC results in deficits in speed of processing information, cognitive flexibility, recall of relevant information and enhanced dopamine activity^49^,^50^. In this respect, it is of interest to note that GABAergic interneurons in the mPFC receive direct input from mesocortical dopaminergic fibers, and this control matures during adolescence and appears crucial for cortical network activity at adulthood^51^. Moreover, together with the genetic background and environmental stress the developmental ingrowth of dopaminergic fibers may lead to an abnormal functioning of the GABAergic interneurons at adulthood, especially under stressful conditions that also alter the dopamine system^52^. A functional dysregulation of the mesostriatal dopaminergic neurons in schizophrenia has indeed been well established^8^,^53^,^54^.

Given that we found altered levels of GAD67 in the mPFC of APO-SUS rats, indicative of a reduced inhibitory drive, we studied changes in the basal synaptic input connectivity of LII/III pyramidal neurons. Surprisingly, mIPSC amplitude and frequency on these neurons were unaltered, as was the presynaptic release probability for GABA, suggesting that the changes in interneuron composition were not accompanied by changes in basal synaptic input. Additionally, we did not observe any changes in the response to bath application of GABA, indicating that the total number of GABA_A_ receptors was unaltered. Together, these results suggest that the total number and strength of inhibitory synapses are not different between APO-SUS and APO-UNSUS rats. However, this study does not include a measure of excitability of the individual classes of interneurons within the mPFC and it is conceivable that the reduced inhibitory activity is due to a reduced excitability of one or more subclasses of interneurons. The use of reporter rodent lines will allow the targeted exploration of the excitability of specific interneuron subclasses in future studies.

While we did not observe differences at the level of synaptic transmission and morphology of LII/III pyramidal cells, we found a clear reduction in the paired-pulse ratio in the APO-SUS compared to APO-UNSUS rats. This reduction was specific for ISIs ≥150 ms, and was not observed in ≤100 ms ISIs. The reduced paired-pulse ratio at ≥150 ms ISIs indicates a reduced release of neurotransmitter at the second pulse. We show that enhanced GABA_B_ receptor signaling, at least in part, underlies this reduction. Indeed, the paired-pulse ratio in the APO-SUS rats could be restored to the levels observed in the APO-UNSUS rats by application of the GABA_B_ receptor antagonist CGP 55845. GABA_B_ receptors are metabotropic G-protein-coupled GABA receptors, and are located perisynaptically at both the pre- and postsynapse^55^. Presynaptic GABA_B_ receptors reduce calcium inflow mainly by inhibiting N- type and P/Q type voltage-gated calcium channels and to a lesser extent by activating inward-rectifying potassium channels such as Kir3, causing a hyperpolarization^55^. Both pathways reduce the inflow of calcium, and occur with a delay of ~150 ms and slow decay of ~1 s^40^ consistent with the maximal effect that we observed at the 150 ms ISI. Postsynaptic GABA_B_ receptors mediate opening of potassium channels, but both the absence of an outward current in our recordings as well as the use of a cesium-based intracellular recording solution exclude the possibility of a postsynaptic contribution of GABA_B_ receptors to our recordings and point to presynaptic GABA_B_ receptor activity underlying the observed changes. Activation of presynaptic GABA_B_ receptors reduces calcium conductance and subsequent GABA release, and therefore provides a negative feedback to the GABAergic system^56^,^57^. Interestingly, a reduced inhibitory input onto excitatory pyramidal cells has been found in models for schizophrenia^20^ and autism^58^, and underlines the notion that a proper tuning of excitation and inhibition is required for proper brain function^11^. Importantly, a number of studies have highlighted a crucial link between GABA transmission and cognitive dysfunction in schizophrenia, indicating that reduced prefrontal inhibitory transmission induces various cognitive, emotional and dopaminergic abnormalities that resemble aspects of this disorder^48^.

Consistent with our finding of increased GABA_B_ signaling, we show that the expression of GABA_B1_ is increased in the mPFC of APO-SUS compared to APO-UNSUS rats. However, our results do not show if the increase in GABA_B1_ is due to an increase in the number of GABA_B1_-expressing cells, or an increase in the amount of GABA_B1_ per cell. Thus far, no comparative studies are available describing the levels of GABA_B_ receptors in individual interneuron subtypes. Interestingly, a reduction in GABA_B_ subunit expression has been observed in various brain regions of schizophrenia patients^59^,^60^,^61^. In humans, stimulation of cortical GABA_B_ receptors in the fronto-parietal network has led to better attentional allocation in reinforcement learning^62^. In addition, GABA_B_ receptor manipulation has been shown to reverse behavioral changes related to psychosis^63^, improve pre-pulse inhibition deficits and ameliorate sensorimotor gating in rodent models^64^. Combining the results of the present animal study with the results of the previously reported patient studies suggests that both reduced as well as increased GABA_B_ signaling may underlie some of the aspects of schizophrenia. Of note, most schizophrenia research on patients is hampered by the use of medication, whereas the rat model used in this study is drug naive. Additional preclinical studies are warranted to further evaluate the hypothesis that the GABA_B_ receptor represents a promising pharmacological target for treating appropriately stratified subsets of individuals with schizophrenia.

It is important to notice that schizophrenia is a disorder with an onset during adolescence or early adulthood^65^. The PND 20–22 rats used for this study are in their early adolescence, and therefore do not necessarily display the schizophrenia-relevant features that have been described in adult rats^21^,^22^,^23^,^24^,^25^,^26^. Even though the exact mechanisms underlying neurodevelopmental disorders, including schizophrenia, are unknown, many theories exist about a distorted balance between neuronal excitation and inhibition during development^51^,^66^,^67^. While it is difficult to distinguish primary effects from homeostatic- and compensatory mechanisms, a substantial amount of evidence points in the direction of disrupted inhibitory signaling as an important factor in the etiology of schizophrenia^51^,^68^,^69^. Our data suggest a possible role for GABA_B_ receptor signaling in the development of the schizophrenia-relevant features observed in adult APO-SUS rats.

In conclusion, our findings highlight the importance of GABAergic signaling for inducing schizophrenia-related phenotypes, and identify GABA_B_ receptors as potential new key players in the distorted network functioning in this disorder.

## Methods

### Animals

All experiments were performed with male Wistar (PND20–22) rats pharmacogenetically selected for high susceptibility (APO-SUS) or low susceptibility (APO-UNSUS) to apomorphine. The generation of APO-SUS and APO-UNSUS rat lines has been described previously^21^. In short, upon injection of apomorphine, a bimodal distribution of the gnawing response was found, i.e. approximately 40% of the Wistar rats showed a weak gnawing response (<10 counts/45 min) and a similar percentage showed an intense gnawing response (>500 counts/45 min). Following this initial selection, the nine males and females with the highest scores, and the nine males and females with the lowest scores were paired and their offspring was again tested for their gnawing response. For each new generation, nine new pairs of rats were selected out of the four male and female litters showing the highest (APO-SUS) and the lowest (APO-UNSUS) mean gnawing response per gender, with the condition that brother/sister pairing was not allowed. APO-SUS rats are defined as animals born from an APO-SUS mother and father; APO-UNSUS rats are likewise defined as animals born from an APO-UNSUS mother and father. The present experiments were performed with naive male APO-SUS and APO-UNSUS rats belonging to the 38^th^ and 40^th^ generation, i.e. apomorphine was used only during the procedure to select and generate the APO-SUS and APO-UNSUS lines.

All rats were bred and reared in the Central Animal Facility of the Radboud University Nijmegen. The animals were reared and housed in macrolon cages (42 × 26 × 15 cm) in a controlled (20 ± 2 °C) and enriched environment under a 12 h light/dark cycle (lights on at 7:00 A.M.). Food pellets and water were provided ad libitum. Experimental procedures were performed between 9:00 A.M. and 5:00 P.M. The experimental procedures were approved by the Animal Ethics Committee of the Radboud University Nijmegen (Nijmegen, the Netherlands) and conducted in accordance with the Dutch legislation. Every effort was made to minimize the number of animals used and their suffering.

### Western blotting

For the western blot experiments the animals were decapitated and the brains were collected quickly, frozen on dry ice, and kept at −80 °C. The brains were sliced into 200 μm coronal sections using a cryostat (Microm HM 500OM) at −15 °C and mounted on glass slides. Punches from 6 sections of the mPFC (prelimbic and infralimbic region) were taken with a 2 mm diameter micropunch needle (Harris Inc.) between the first appearance of the corpus callosum and the start of the caudate putamen. The coordinates were determined according to the atlas of Paxinos and Watson^70^. Samples from each hemisphere were pooled. All the samples were stored at −80 °C before protein extraction took place.

Brain punches were homogenized in 50 μl of lysis buffer (50 mM Hepes pH 7.4, 140 mM NaCl, 0.1% Triton-X100, 1% Tween 20, 0.1% deoxycholate) supplemented with protease inhibitor mix (Roche Diagnostics). The protein levels were assessed using the Bradford assay. After protein measurement, sodium dodecyl sulfate polyacrilamide gel electrophoresis (SDS-PAGE) on 10% (w/v) at 100 V for 90 minutes was carried out using a Mini-Protean system (Bio-Rad, USA). Protein (40 μg) was loaded in each lane with loading buffer (0.25 M Tris-HCl, pH 6.8, 2% SDS, 10% glycerol, 0.25% bromophenolblue, 4% beta-mercaptoethanol). After electrophoresis, proteins were transferred to a polyvinylidene fluoride membrane (PVDF, Amersham, Hybond-P), using an electrophoretic transfer system (Bio-Rad, USA) at 400 mA for 90 minutes. The membranes were then blocked with 5% skimmed milk dissolved in 0.1 M PBS for one hour. The membranes were incubated overnight at 4 °C with the primary antibodies diluted in blocking buffer containing 5% skimmed milk dissolved in a PBS Tween 20 mixture (5%) (PBS-T, Sigma-Aldrich). The primary antibodies were the following: mouse monoclonal antibodies for CB D-28K (1:1000, Swant), CR (1:1000, Swant), GAD67 (1:1000, Abcam), GAT1 (1:500, Abcam), GABAB1 (1:500, Abcam), and gamma-tubulin (1:10000, Sigma). After being washed for one hour with 1% skimmed milk in PBS-T (0.05%), the membranes were incubated for one hour in the dark at room temperature with goat-anti-mouse secondary antibody (1:10.000; LI-COR Biosciences Inc; IRDye 680). The membranes were imaged using an Odyssey IR-scanner (LI-COR Biosciences, Inc.) and the generated pictures were quantified using the Odyssey software (Li-COR Biosciences Inc.). The levels of protein expression were normalized to beta-tubulin. Protein expression values are normalized to APO-UNSUS values (relative intensity).

### Immunohistochemistry

For the fluorescent immunohistochemistry experiments, rats were sacrificed by direct decapitation without anesthesia, after which the brains were extracted and post-fixated by immersion in 2% paraformaldehyde (PFA) in 0.1 M PBS, pH 7.4 for 48 hours. After post-fixation, the brains were transferred to a 30% sucrose solution for cryoprotection. Coronal sections (10 μm) were cut on cryostate, collected on SuperFrost glass slides and stored at −20 °C until further processing.

Sections (10 μm) were incubated for 1 h in blocking buffer (5% normal goat, horse and donkey serum, 1% BSA, 1% glycine, 0,1% lysine, 0,4% Triton X-100) to minimize nonspecific labeling. Tissue sections were then incubated overnight at room temperature with mouse monoclonal primary antibodies diluted in blocking buffer against CB D-28k (1:2000; CB300; Swant), CR (1:3500; CR-6B3, Swant), or PV (1:8000; PV-235, Swant); for GAD67 (1:5000; K-87, Abcam), incubation was done for 4 h at room temperature, followed by a 65 h incubation at 4 °C. The next morning, sections were washed in PBS and placed for 1.5 h in a 1:500 dilution of goat-anti-mouse Alexa-fluor 488 secondary antibody (Invitrogen). Hoechst (bisBenzimide dye, No 33342, Sigma B-2261; 1 μg/mL prepared in 0.1 M PBS) staining was performed for nuclear staining. After overnight drying of the sections at room temperature, they were coverslipped with FluorSaveTM Reagent (Merck Millipore). The next day, slides were visualized with an automated high content epifluorescence microscope with digital image acquisition (Leica DMI6000B inverted microscope, Leica EL6000 illumination source). The pictures were manually analyzed and quantified at 10X magnification using FIJI3 in a blinded fashion. The values obtained for each animal represent the average of measurements taken from 3–4 equally spaced sections for each brain area. Nomenclature of the brain was based on the atlas of Paxinos and Watson^70^.

### Electrophysiology

Rats were anesthetized with isoflurane before decapitation. Coronal slices (350 μm) were cut using a HM650V vibration microtome (Thermo Scientific) in ice cold artificial cerebrospinal fluid (ACSF) containing (in mM): 124 NaCl; 11 Glucose; 3 KCl; 1.25 NaH_2_PO_4_; 1 CaCl_2_; 4 MgCl_2_; 26 NaHCO_3_, continuously oxygenated with 95% O_2_/5% CO_2_ and incubated for 1 h at room temperature. Slices were transferred to the recording setup 15 minutes prior to recording and incubated at 30 °C while being continuously oxygenated with 95% O_2_/5% CO_2_ in recording ACSF containing (unless otherwise stated)(in mM): 124 NaCl, 1.25 NaH_2_PO_4_, 3 KCl, 26 NaHCO_3_, 10 Glucose, 2 CaCl_2_, 1 MgCl_2_. Patch pipettes (3–5 MΩ) were made from borosilicate glass capillaries and filled with intracellular solution containing (in mM): 115 CsMeSO_3_; 20 CsCl; 10 HEPES; 2.5 MgCl_2_; 4 Na_2_ATP; 0.4 NaGTP; 10 Na-Phosphocreatine; 0.6 EGTA. Traces were recorded using a Multiclamp 700B amplifier (Molecular Devices), sampled at 10 kHz and filtered at 2 kHz. Cells were excluded from analysis if the series resistance exceeded 25 MΩ. mIPSCs were recorded in the presence of Tetrodotoxin (TTX, 1 µM), 6-cyano-7-nitroquinoxaline-2,3-dione (CNQX, 5 µM) and (2*R*)-amino-5-phosphonovaleric acid (APV, 100 µM) at a holding potential of +10 mV. mEPSC were recorded in the presence of TTX and Picrotoxin (PTX, 100 µM) at a holding potential of −60 mV. Paired-pulse ratio (PPR) was recorded following stimulation in LII/III of the PFC in the presence of CNQX and D-APV, in ACSF containing 4 mM CaCl_2_ and 4 mM MgCl_2_ to increase the stimulated responses, at a holding potential of −60 mV. Stimulation strength was set to evoke a ~200 pA response to the first stimulus. Two 1 ms pulses were given with a 50 ms, 100 ms, 150 ms, 200 ms or 500 ms interval. PPR was calculated as peak2/peak1 after correcting for any residual current at the second pulse. CGP 55845 was used at a concentration of 10 μM^71^. For GABA application, cells were allowed to stabilize their holding current, after which 20 μM GABA was washed in. We quantified the maximal change in holding current as the maximal response to the washed in GABA. Miniature recordings were analyzed using Mini Analysis Program (Synaptosoft). Other traces were analyzed using Clampfit 10.2. All drugs were purchased from Tocris.

### Morphological reconstructions

For morphological reconstructions, the internal solution was supplemented with 0.4% biocytin (Sigma-Aldrich). Following single-cell electrophysiology, the slices with biocytin-filled neurons were fixated in 4% paraformaldehyde in 0.1 M PBS overnight at 4 °C, and subsequently processed following a modified staining protocol based on Marx *et al*.^72^. In brief: after fixation, slices were rinsed in 0.1 M PB, incubated in 3% H2O2 in 0.1 M PB for 30 minutes at room temperature to quench endogenous peroxidases, rinsed in 0.1 M PBS, then incubated in Avidin-Biotin-Peroxidase solution (Vectastain Elite, with 1% v/v Triton-100) overnight on a shaking platform at 4 °C. The next day, slices were washed with 0.1 M PBS and pre-incubated with Di-Aminobenzidine (DAB) solution with Nickel enhancer (Vector Peroxidase substrate kit, SK-4100) for 30 min. Then, the DAB solution was replaced with the same solution plus H2O2 and incubated for ca. 30 seconds. Slices were then rinsed in 0.1 M PB, mounted on gelatinized coverslips, and dried for 3–6 h in a custom-made moist chamber at room temperature. Slices were dehydrated in an ethanol series and Xylene, put on coverslips and sealed with Eukitt (Sigma). Slices were imaged on a Zeiss Axioskop 1 upright brightfield microscope with 20x and 40x objectives (Zeiss EC Plan-Neofluar, NA 0.5 and 0.75, respectively) and motorized stage (MicroBrightField). The camera and motorized stage were connected to a Neurolucida workstation (MicroBrightField). Cells were selected based on position in the cortical layer II/III of the prefrontal cortex, pyramidal morphology, and staining intensity. Somata, apical and basal dendrites were reconstructed in Neurolucida as three-dimensional drawings. Reconstructions were analysed in NeuroExplorer (MicroBrightField) for intrinsic parameters and Sholl analysis.

### Statistical analysis

Statistical analysis was performed using Prism (Graphpad). Significance was tested with a two-sided Student’s t-test. Correction for multiple comparisons was performed using the Holm-Sidak method where indicated. Data is expressed as mean ± SEM. Significance was defined as p < 0.05 (*) or p < 0.01 (**).

## Additional Information

**How to cite this article**: Selten, M. M. *et al*. Increased GABA_B_ receptor signaling in a rat model for schizophrenia. *Sci. Rep.* **6**, 34240; doi: 10.1038/srep34240 (2016).

## Figures and Tables

**Table 1:** Dendritic morphological analysis of reconstructed layer II/III pyramidal cells.

Morphology		UNSUS	SUS	p-value	Sign
Basal Dendrites	Quantity	6.0 ± 0.9	9.3 ± 1.4	0.029	n.s.
	Nodes	18.5 ± 2.1	20.8 ± 2.5	0.404	n.s.
	Ends	24.3 ± 2.6	29.8 ± 1.8	0.060	n.s.
	Length (μm)	2339.9 ± 407.5	2659.7 ± 189.1	0.433	n.s.
	Mean length	397.2 ± 59.1	312.8 ± 54.4	0.224	n.s.
Apical Dendrite	Nodes	15.0 ± 1.7	22.0 ± 4.0	0.093	n.s.
	Ends	16.0 ± 1.7	23.0 ± 4.0	0.093	n.s.
	Length (μm)	1775.9 ± 183.1	2617.7 ± 490.4	0.085	n.s.
	Max Branch Order	8.0 ± 0.5	11.0 ± 0.8	0.009	n.s.
Convex Hull	Area (μm^2^)	43375.2 ± 2899.2	55194.9 ± 8447.3	0.143	n.s.
	Perimeter (μm)	831.0 ± 20.8	929.5 ± 66.6	0.116	n.s.

Student’s t-test with Holm-Sidak correction for multiple comparisons. Data is presented as mean ± SEM. Sign: significance; n.s: not significant.

## References

[b1] Sullivan P. F., Kendler K. S. & Neale M. C. Schizophrenia as a Complex Trait. Arch. Gen. Psychiatry 60, 1187–1192 (2003). 14662550 10.1001/archpsyc.60.12.1187

[b2] Tsuang M. T., Bar J. L., Stone W. S. & Faraone S. V. Gene-environment interactions in mental disorders. World Psychiatry 3, 73–83 (2004). 16633461 PMC1414673

[b3] Burrows E. L. & Hannan A. J. Decanalization mediating gene-environment interactions in schizophrenia and other psychiatric disorders with neurodevelopmental etiology. Front. Behav. Neurosci. 7, 1–5 (2013). 24312026 10.3389/fnbeh.2013.00157 PMC3826253

[b4] Lawrie S. M. . Reduced frontotemporal functional connectivity in schizophrenia associated with auditory hallucinations. Biol. Psychiatry 51, 1008–1011 (2002). 12062886 10.1016/s0006-3223(02)01316-1

[b5] Stephan K. E., Friston K. J. & Frith C. D. Dysconnection in Schizophrenia: From abnormal synaptic plasticity to failures of self-monitoring. Schizophr. Bull. 35, 509–527 (2009). 19155345 10.1093/schbul/sbn176 PMC2669579

[b6] Meyer-Lindenberg A. From maps to mechanisms through neuroimaging of schizophrenia. Nature 468, 194–202 (2010). 21068827 10.1038/nature09569

[b7] Fornito A., Yoon J., Zalesky A., Bullmore E. T. & Carter C. S. General and specific functional connectivity disturbances in first-episode schizophrenia during cognitive control performance. Biol. Psychiatry 70, 64–72 (2011). 21514570 10.1016/j.biopsych.2011.02.019 PMC4015465

[b8] Kuepper R., Skinbjerg M. & Abi-Dargham A. In Current Antipsychotics 1–26 (2012). 10.1007/978-3-642-25761-2_1 23129326

[b9] Bhattacharyya S., Raote I., Bhattacharya A., Miledi R. & Panicker M. M. Activation, internalization, and recycling of the serotonin 2A receptor by dopamine. Proc. Natl. Acad. Sci. USA 103, 15248–15253 (2006). 17005723 10.1073/pnas.0606578103 PMC1622808

[b10] Moghaddam B. & Javitt D. From Revolution to Evolution: The Glutamate Hypothesis of Schizophrenia and its Implication for Treatment. Neuropsychopharmacology 37, 4–15 (2012). 21956446 10.1038/npp.2011.181 PMC3238069

[b11] Marín O. Interneuron dysfunction in psychiatric disorders. Nat. Rev. Neurosci. 107–120, doi: 10.1038/nrn3155 (2012). 22251963

[b12] Del Pino I. . Erbb4 deletion from fast-spiking interneurons causes schizophrenia-like phenotypes. Neuron 79, 1152–1168 (2013). 24050403 10.1016/j.neuron.2013.07.010

[b13] Beasley C. L., Zhang Z. J., Patten I. & Reynolds G. P. Selective deficits in prefrontal cortical GABAergic neurons in schizophrenia defined by the presence of calcium-binding proteins. Biol. Psychiatry 52, 708–715 (2002). 12372661 10.1016/s0006-3223(02)01360-4

[b14] Reynolds G. P., Abdul-Monim Z., Reynolds G. P., Zuhal Neill J. C. & Zhang Z. Calcium Binding Protein Markers of GABA Deficits in Schizophrenia - Post Mortem Studies and Animal Models. Neurotox. Res. 6, 57–61 (2004). 15184106 10.1007/BF03033297

[b15] Sakai T. . Changes in density of calcium-binding-protein-immunoreactive GABAergic neurons in prefrontal cortex in schizophrenia and bipolar disorder. Neuropathology 28, 143–150 (2008). 18069969 10.1111/j.1440-1789.2007.00867.x

[b16] Schleimer S. B., Hinton T., Dixon G. & Johnston G. A. R. GABA transporters GAT-1 and GAT-3 in the human dorsolateral prefrontal cortex in schizophrenia. Neuropsychobiology 50, 226–230 (2004). 15365220 10.1159/000079975

[b17] Hashimoto T. . Gene expression deficits in a subclass of GABA neurons in the prefrontal cortex of subjects with schizophrenia. J. Neurosci. 23, 6315–6326 (2003). 12867516 10.1523/JNEUROSCI.23-15-06315.2003 PMC6740534

[b18] Lazarus M. S., Krishnan K. & Huang Z. J. GAD67 Deficiency in Parvalbumin Interneurons Produces Deficits in Inhibitory Transmission and Network Disinhibition in Mouse Prefrontal Cortex. Cereb. Cortex 1290–1296, doi: 10.1093/cercor/bht322 (2013). PMC4481616 24275833

[b19] Volk D. W. . Reciprocal alterations in pre- and postsynaptic inhibitory markers at chandelier cell inputs to pyramidal neurons in schizophrenia. Cereb. Cortex 12, 1063–1070 (2002). 12217970 10.1093/cercor/12.10.1063

[b20] Fazzari P. . Control of cortical GABA circuitry development by Nrg1 and ErbB4 signalling. Nature 464, 1376–1380 (2010). 20393464 10.1038/nature08928

[b21] Cools A. R., Brachten R., Heeren D., Willemen A. & Ellenbroek B. Search after neurobiological profile of individual-specific features of Wistar rats. Brain Res. Bull. 24, 49–69 (1990). 2310946 10.1016/0361-9230(90)90288-b

[b22] Rots N. Y. . Rats bred for enhanced apomorphine susceptibility have elevated tyrosine hydroxylase mRNA and dopamine D2-receptor binding sites in nigrostriatal and tuberoinfundibular dopamine systems. Brain Res. 710, 189–196 (1996). 8963658 10.1016/0006-8993(95)01379-2

[b23] Cools A. R., Ellenbroek B. A., Gingras M. A., Engbersen A. & Heeren D. Differences in vulnerability and susceptibility to dexamphetamine in Nijmegen high and low responders to novelty: A dose-effect analysis of spatio-temporal programming of behaviour. Psychopharmacology (Berl). 132, 181–187 (1997). 9266615 10.1007/s002130050334

[b24] Van der Elst M. C. J., Ellenbroek B. A. & Cools A. R. Cocaine strongly reduces prepulse inhibition in apomorphine-susceptible rats, but not in apomorphine-unsusceptible rats: Regulation by dopamine D2 receptors. Behav. Brain Res. 175, 392–398 (2006). 17079027 10.1016/j.bbr.2006.09.014

[b25] Ellenbroek B. A., Geyer M. A. & Cools A. R. The behavior of APO-SUS rats in animal models with construct validity for schizophrenia. J. Neurosci. 15, 7604–7611 (1995). 7472511 10.1523/JNEUROSCI.15-11-07604.1995 PMC6578060

[b26] Van Der Elst M. C. J. . A single exposure to novelty differentially affects the accumbal dopaminergic system of apomorphine-susceptible and apomorphine-unsusceptible rats. Life Sci. 76, 1391–1406 (2005). 15670618 10.1016/j.lfs.2004.10.023

[b27] Cools A. R. . Apomorphine-susceptible and apomorphine-unsusceptible Wistar rats differ in novelty-induced changes in hippocampal dynorphin B expression and two-way active avoidance: A new key in the search for the role of the hippocampal- accumbens axis. Behav. Brain Res. 55, 213–221 (1993). 8102850 10.1016/0166-4328(93)90117-9

[b28] Tuinstra T. . Retrieval of spatial information in Nijmegen high and low responders: involvement of beta-adrenergic mechanisms in the nucleus accumbens. Behav. Neurosci. 114, 1088–1095 (2000). 11142641 10.1037//0735-7044.114.6.1088

[b29] Daviss S. R. & Lewis D. A. Local circuit neurons of the prefrontal cortex in schizophrenia: selective increase in the density of calbindin-immunoreactive neurons. Psychiatry Res. 59, 81–96 (1995). 8771223 10.1016/0165-1781(95)02720-3

[b30] Lund J. S. & Lewis D. A. Local circuit neurons of developing and mature macaque prefrontal cortex: Golgi and immunocytochemical characteristics. J. Comp. Neurol. 328, 282–312 (1993). 7678612 10.1002/cne.903280209

[b31] Lau C. G. & Murthy V. N. Activity-dependent regulation of inhibition via GAD67. J. Neurosci. 32, 8521–8531 (2012). 22723692 10.1523/JNEUROSCI.1245-12.2012 PMC3388776

[b32] Jiao Y., Zhang C., Yanagawa Y. & Sun Q.-Q. Major Effects of Sensory Experiences on the Neocortical Inhibitory Circuits. J. Neurosci. 26, 8691–8701 (2006). 16928857 10.1523/JNEUROSCI.2478-06.2006 PMC6674381

[b33] Okada M., Onodera K., Van Renterghem C., Sieghart W. & Takahashi T. Functional correlation of GABA(A) receptor alpha subunits expression with the properties of IPSCs in the developing thalamus. J. Neurosci. 20, 2202–2208 (2000). 10704495 10.1523/JNEUROSCI.20-06-02202.2000 PMC6772493

[b34] Dunning D. D., Hoover C. L., Soltesz I., Smith M. A. & Dowd D. K. O. GABA A Receptor − Mediated Miniature Postsynaptic Currents and α -Subunit Expression in Developing Cortical Neurons. J. Neurophysiol. 3286–3297 (2014). 10.1152/jn.1999.82.6.3286 10601460

[b35] Van Aerde K. I. & Feldmeyer D. Morphological and Physiological Characterization of Pyramidal Neuron Subtypes in Rat Medial Prefrontal Cortex. Cereb. Cortex 788–805, doi: 10.1093/cercor/bht278 (2013). 24108807

[b36] Caillard O., McLean H. A., Ben-ari Y. & Gaïarsa J. Ontogenesis of Presynaptic GABAB Receptor-Mediated Inhibition in the CA3 Region of the Rat Hippocampus 1341–1348 (1998). 10.1152/jn.1998.79.3.1341 9497415

[b37] Volgushev M., Balaban P., Chistiakova M. & Eysel U. T. Retrograde signalling with nitric oxide at neocortical synapses. Eur. J. Neurosci. 12, 4255–4267 (2000). 11122337 10.1046/j.0953-816x.2000.01322.x

[b38] Wilson R. I. & Nicoll R. A. Endogenous cannabinoids mediate retrograde signalling at hippocampal synapses. Nature 410, 2–6 (2001). 10.1038/35069076 11279497

[b39] Olpe H.-R., Steinmann M. W., Greiner K. & Pozza M. F. Contribution of presynaptic GABA-B receptors to paired-pulse depression of GABA-responses in the hippocampus. 473–477 (1994). 10.1007/BF00169135 8065460

[b40] Chalifoux J. R. & Carter A. G. GABA B Receptor Modulation of Voltage-Sensitive Calcium Channels in Spines and Dendrites. 31, 4221–4232 (2011). 10.1523/JNEUROSCI.4561-10.2011 PMC3061967 21411663

[b41] Gonzalez-Burgos G., Rotaru D. C., Zaitsev A. V., Povysheva N. V. & Lewis D. A. GABA transporter GAT1 prevents spillover at proximal and distal GABA synapses onto primate prefrontal cortex neurons. J. Neurophysiol. 101, 533–547 (2009). 19073797 10.1152/jn.91161.2008 PMC2657080

[b42] Volk D. W., Austin M. C., Pierri J. N., Sampson A. R. & Lewis D. A. GABA transporter-1 mRNA in the prefrontal cortex in schizophrenia: Decreased expression in a subset of neurons. Am. J. Psychiatry 158, 256–265 (2001). 11156808 10.1176/appi.ajp.158.2.256

[b43] Jones K. . GABA(B) receptors function as a heteromeric assembly of the subunits GABA(B)R1 and GABA(B)R2. Nature 396, 674–679 (1998). 9872315 10.1038/25348

[b44] Nakazawa K. . GABAergic interneuron origin of schizophrenia pathophysiology. Neuropharmacology 62, 1574–1583 (2012). 21277876 10.1016/j.neuropharm.2011.01.022 PMC3090452

[b45] Pocklington A. J. . Novel Findings from CNVs Implicate Inhibitory and Excitatory Signaling Complexes in Schizophrenia. Neuron 86, 1203–1214 (2015). 26050040 10.1016/j.neuron.2015.04.022 PMC4460187

[b46] Guidotti A. . Decrease in Reelin and Glutamic Acid Decarboxylase 67 (GAD 67) Expression in Schizophrenia and Bipolar Disorder. Arch. Gen. Psychiatry 57, 1061–1069 (2000). 11074872 10.1001/archpsyc.57.11.1061

[b47] Akbarian S. & Huang H. S. Molecular and cellular mechanisms of altered GAD1/GAD67 expression in schizophrenia and related disorders. Brain Res. Rev. 52, 293–304 (2006). 16759710 10.1016/j.brainresrev.2006.04.001

[b48] Tse M. T., Piantadosi P. T. & Floresco S. B. Prefrontal Cortical Gamma-Aminobutyric Acid Transmission and Cognitive Function: Drawing Links to Schizophrenia from Preclinical Research. Biol. Psychiatry 77, 929–939 (2015). 25442792 10.1016/j.biopsych.2014.09.007

[b49] Enomoto T., Tse M. T. & Floresco S. B. Reducing prefrontal gamma-aminobutyric acid activity induces cognitive, behavioral, and dopaminergic abnormalities that resemble schizophrenia. Biol. Psychiatry 69, 432–441 (2011). 21146155 10.1016/j.biopsych.2010.09.038

[b50] Piantadosi P. T. & Floresco S. B. Prefrontal cortical GABA transmission modulates discrimination and latent inhibition of conditioned fear: Relevance for schizophrenia. Neuropsychopharmacology 39, 2473–2484 (2014). 24784549 10.1038/npp.2014.99 PMC4138759

[b51] O’Donnell P. Adolescent onset of cortical disinhibition in schizophrenia: Insights from animal models. Schizophr. Bull. 37, 484–492 (2011). 21505115 10.1093/schbul/sbr028 PMC3080677

[b52] Benes F. M. & Berretta S. GABAergic interneurons: implications for understanding schizophrenia and bipolar disorder. Neuropsychopharmacology 25, 1–27 (2001). 11377916 10.1016/S0893-133X(01)00225-1

[b53] Laruelle M., Frankle W. G., Narendran R., Kegeles L. S. & Abi-Dargham A. Mechanism of action of antipsychotic drugs: From dopamine D2 receptor antagonism to glutamate NMDA facilitation. Clin. Ther. 27 (2005). 10.1016/j.clinthera.2005.07.017 16198197

[b54] Carlsson A. . Interactions Between Monoamines, Glutamate, and GABA in Schizophrenia: New Evidence. Annu. Rev. Pharmacol. Toxicol 63, 108–116 (2001). 10.1146/annurev.pharmtox.41.1.237 11264457

[b55] Benarroch E. E. GABAB receptors: structure, functions, and clinical implications. Neurology 78, 578–584 (2012). 22351795 10.1212/WNL.0b013e318247cd03

[b56] Davies C. H., Starkey S. J., Pozza M. F. & Collingridge G. L. GABAB autoreceptors regulate the induction of LTP. Nature 349, 609–611 (1991). 1847993 10.1038/349609a0

[b57] Leung L. S. & Peloquin P. GABAB receptors inhibit backpropagating dendritic spikes in hippocampal CA1 pyramidal cells *in vivo*. Hippocampus 16, 388–407 (2006). 16411229 10.1002/hipo.20168

[b58] Gibson J. R., Huber K. M. & Südhof T. C. Neuroligin-2 deletion selectively decreases inhibitory synaptic transmission originating from fast-spiking but not from somatostatin-positive interneurons. J. Neurosci. 29, 13883–13897 (2009). 19889999 10.1523/JNEUROSCI.2457-09.2009 PMC2814361

[b59] Mizukami K. . Immunohistochemical localization of gamma-aminobutyric acid(B) receptor in the hippocampus of subjects with schizophrenia. Neurosci. Lett. 283, 101–104 (2000). 10739885 10.1016/s0304-3940(00)00939-3

[b60] Mizukami K. . Immunohistochemical localization of GABA B receptor in the entorhinal cortex and inferior temporal cortex of schizophrenic brain. Prog. Neuropsychopharmacol. Biol. Psychiatry 26, 393–396 (2002). 11817519 10.1016/s0278-5846(01)00247-0

[b61] Fatemi S. H., Folsom T. D. & Thuras P. D. Deficits in GABAB receptor system in schizophrenia and mood disorders: A postmortem study. Schizophr. Res. 128, 37–43 (2011). 21303731 10.1016/j.schres.2010.12.025 PMC3085603

[b62] Ort A., Kometer M., Rohde J., Seifritz E. & Vollenweider F. X. The role of GABAB receptors in human reinforcement learning. Eur. Neuropsychopharmacol. 24, 1606–1614 (2014). 25194227 10.1016/j.euroneuro.2014.08.013

[b63] Wierońska J. M. . The GABA B receptor agonist CGP44532 and the positive modulator GS39783 reverse some behavioural changes related to positive syndromes of psychosis in mice. Br. J. Pharmacol. 163, 1034–1047 (2011). 21371011 10.1111/j.1476-5381.2011.01301.x PMC3130949

[b64] Frau R. . Positive Allosteric Modulation of GABAB Receptors Ameliorates Sensorimotor Gating in Rodent Models. CNS Neurosci. Ther. 20, 679–684 (2014). 24703381 10.1111/cns.12261 PMC4062587

[b65] Fatemi S. H. & Folsom T. D. The neurodevelopmental hypothesis of Schizophrenia, revisited. Schizophr. Bull. 35, 528–548 (2009). 19223657 10.1093/schbul/sbn187 PMC2669580

[b66] Mullins C., Fishell G. & Tsien R. W. Review Unifying Views of Autism Spectrum Disorders: A Consideration of Autoregulatory Feedback Loops. Neuron 89, 1131–1156 (2016). 26985722 10.1016/j.neuron.2016.02.017 PMC5757244

[b67] Nelson S. B. & Valakh V. Excitatory/Inhibitory Balance and Circuit Homeostasis in Autism Spectrum Disorders. Neuron 87, 684–698 (2015). 26291155 10.1016/j.neuron.2015.07.033 PMC4567857

[b68] del Pino I. . Erbb4 Deletion from Fast-Spiking Interneurons Causes Schizophrenia-like Phenotypes. Neuron 79, 1152–1168 (2013). 24050403 10.1016/j.neuron.2013.07.010

[b69] Yu Z. . GABA Transporter-1 Deficiency Confers Schizophrenia-Like Behavioral Phenotypes. PLoS One 8 (2013). 10.1371/journal.pone.0069883 PMC3726734 23922840

[b70] Paxinos G. & Watson C. The Rat Brain in Stereotaxic Coordinates 4th edition (Academic Press, 1998).

[b71] Deidda G. . Reversing excitatory GABAAR signaling restores synaptic plasticity and memory in a mouse model of Down syndrome. Nat. Med. 21, 318–326 (2015). 25774849 10.1038/nm.3827

[b72] Marx M., Günter R. H., Hucko W., Radnikow G. & Feldmeyer D. Improved biocytin labeling and neuronal 3D reconstruction. Nat. Protoc. 7, 394–407 (2012). 22301777 10.1038/nprot.2011.449

